# Tin-Based Antitumour Drugs: New Developments

**DOI:** 10.1155/MBD.1995.99

**Published:** 1995

**Authors:** Marcel Gielen

**Affiliations:** 1 Free University of Brussels VUB Faculty of Applied Sciences Department of General and Organic Chemistry Room 8G512, Pleinlaan 2 Brussel B-1050 Belgium

## Abstract

An overview of the *in vitro* test results on several human tumour cell lines of several series of organotin compounds synthesized at the Free University of Brussels VUB. Several compounds exhibit excellent antitumour activities. *in vivo* screening also gave very promising results.


### TIN-BASED ANTITUMOUR DRUGS: NEW DEVELOPMENTS

Marcel Gielen

Free University of Brussels VUB, Faculty of Applied Sciences,
Department of General and Organic Chemistry, Room 8G512, Pleinlaan 2, B-1050 Brussel, Belgium

**Abstract**

An overview of the *in vitro* test results on several human tumour cell lines of several series of organotin compounds synthesized at the Free University of Brussels VUB. Several compounds exhibit excellent antitumour activities. *In vivo* screening also gave very promising results.

**Antitumour activity of diorganotin 2,6-pyridinedicarboxylates**

Organotin compounds that exhibit promising antitumour properties were synthesized and characterized at the Free University of Brussels. We would like to summarize here the results that have already been patented[1] and that may therefore be disclosed[2].

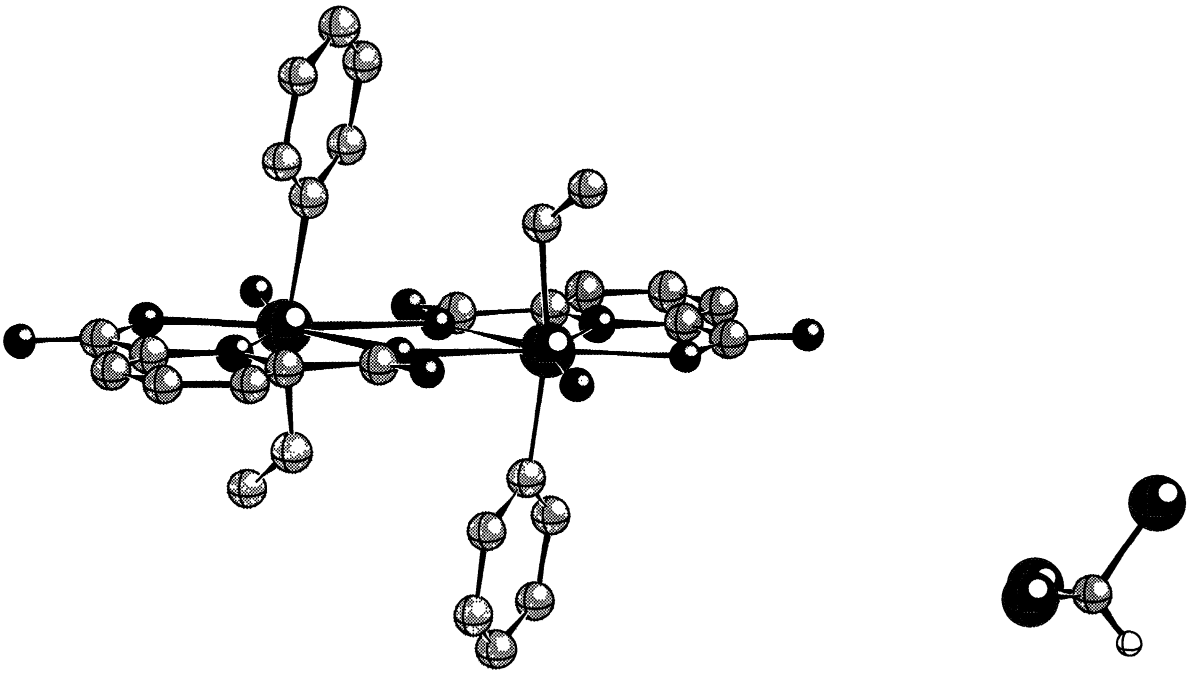

Fig. 1: X-Ray structure of ethylphenyl pyridine-2,6-dicarboxylate.$H_2O$ dimer, $HCCl_3$ solvate[3]

A series of diorganotin 2,6-pyridinedicarboxylates, $C_5H_3N(COO)_2SnRR'$, were prepared and tested[4], with different groups R and R' bound to tin.
The crystal structure of the ethylphenyltin derivative[3] is shown in fig. 1.
The most *in vitro* active compounds are the di-n-butyl ones: they are characterized by inhibition doses $ID_{50}$ of 60 and 106 ng/mL, respectively, against two human cancer cell lines, MCF-7, a breast cancer, and WiDr, a colon carninoma[2]. For cisplatin, the $ID_{50}$ values obtained for the same tumour cell lines are 850 and 624 ng/mL, respectively.

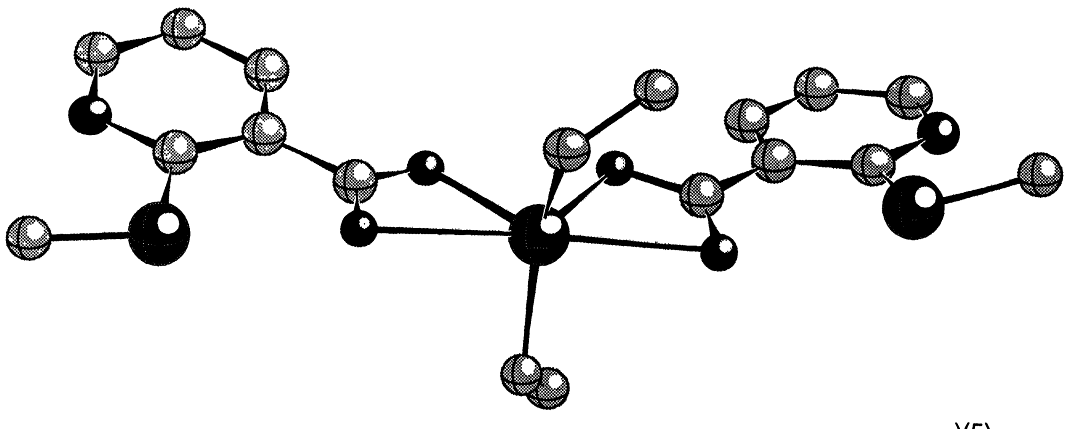

Fig. 2: X-Ray structure of diethyltin bis(2-methylthio-3-pyridinecarboxylate)[5]

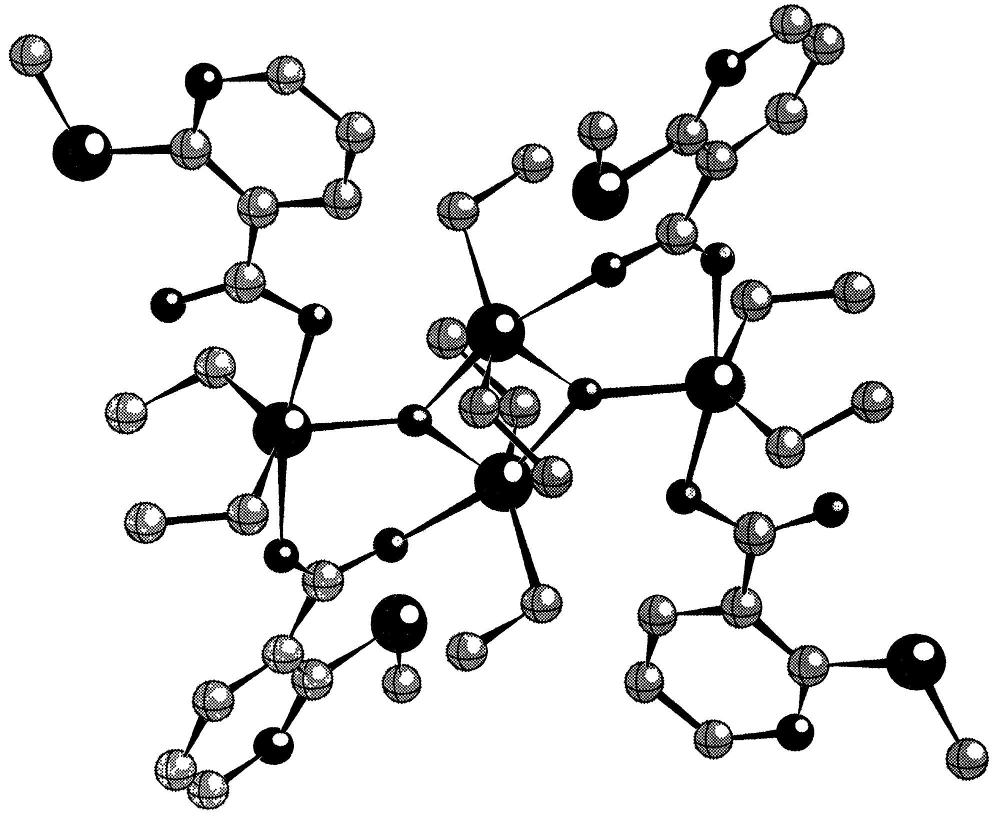

Fig. 3: X-Ray structure of the [diethyl(2-methylthio-3-pyridinecarboxylato)tin oxide] dimer[5]

**Antitumour activity of diorganotin dicarboxylates**

Diorganotin derivatives of substituted salicylic acids[2] were also synthesized. Here, two types of compounds can be made depending on the molar ratio carboxylic acid:diorganotin oxide used.
When a 2:1 molar ratio is used, the expected diorganotin dicarboxylate is formed. The structure of diethyltin bis(2-methylthio-3-pyridinecarboxylate)[5] is shown in fig. 2.
The di-n-butyltin compounds are again the most active ones. For instance, di-n-butyltin bis(4-hydroxy-3-methoxybenzoate) is characterized by $ID_{50}$ values of 44 and 82 ng/mL against MCF-7

and WiDr, respectively[5].

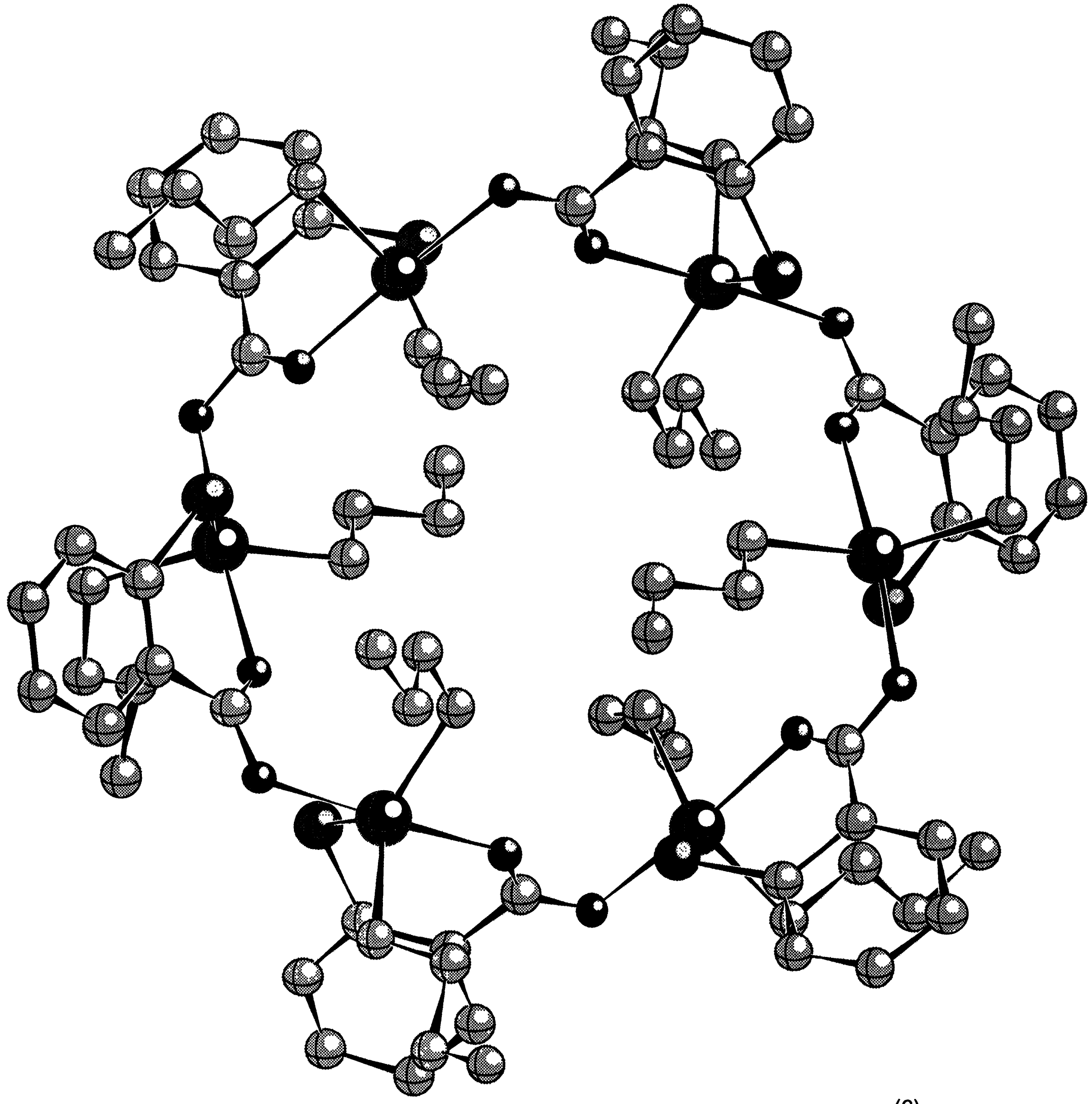

Fig. 4: X-Ray structure of the hexamer of di-n-butyltin thiosalicylate[6]

**Antitumour activity of bis[carboxylato(diorganotin)] oxides**

When a 1:1 molar ratio is used, a dimer of a bis[carboxylato(diorganotin)] oxide is obtained. The crystal structure of [diethyl(2-methylthio-3-pyridinecarboxylato)tin] oxide[5] is shown in fig. 3; this compounds remains a dimer in $CDCl_3$ solution.

Once more, the di-n-butyltin derivatives proved to be the most active ones, much more active than cisplatin: the 1:1 condensation compound di-n-butyltin oxide with 5-methoxysalicylic acid, {[(5-$CH_3OC_6H_3(OH)COO)(Bu_2)Sn]_2O\}_2$, for instance, is characterized by $ID_{50}$ values of 29 and 122 ng/mL against MCF-7 and WiDr, respectively.

In contrast to 1:1 condensation compounds of diorganotin oxides with salicylic acid, that are in fact dimeric distannoxanes in which the phenolic oxygen is not lost, di-n-butyltin thiosalicylate crystallizes as a hexamer[6] (see fig. 4) with tin-carboxylate and tin-sulphur bonds, but becomes monomeric in polar solvents such as DMSO, ethanol or water, in which the drugs are administrated to perform the anticancer screening.

### Exceptionally high antitumour activity of triphenyltin carboxylates

Several series of organotin molecules were prepared that are as active as mitomycin C *in vitro* against MCF-7 and WiDr. The first of these, that have recently been patented[1], are triphenyltin carboxylates $XYZC_6H_2COOSnPh_3$[7].
Several of them (like the 2-methoxybenzoate or the 5-methoxysalicylate), are characterized by $ID_{50}$ values of ca. 15 ng/mL against both MCF-7 and WiDr.
Other series of organotin compounds were synthesized that were found more active than these last ones but they have first to be patented before more can be disclosed about them.

### *In vivo* activity of organotin compounds

The the toxicity profiles *in vivo* in mice and the antitumour activity in tumour-bearing mice were screened[8] for five organotin compounds that were found especially active *in vitro*: triphenyltin 5-sulfosalicylate (**1**), triphenyltin 5-aminosalicylate (**2**), triphenyltin 4-fluorobenzoate (**3**), tri-n-butyltin 2,6-difluorobenzoate (**4**) and di-n-butyltin bis(2,5-dihydroxybenzoate) (**5**).
*In vivo* (table), compound **1** was most toxic mainly through paralysis. At their maximum tolerated dosis (MTD) for a single administration, compounds **1**, **2**, **3** and **4** are inactive against murine colon carcinoma Colon 26. At a single administration, compound **5** was the most active with a Test/Control ratio (T/C) below 0.6 and Growth Delay Factor (GDF) above 1.0, their respective cut-off levels for sensitivity.

Table: Antitumour effect of the tested compounds on Colon 26: Growth Delay Factor GDF, T/C values, Tumour Doubling Time TDT, Median Life Span MLS and Increase inLife Span ILS

| Treatment | GDF | T/C | mean TDT | MLS | ILS |
|---|---|---|---|---|---|
| control | | | | 20 | 100 |
| **1**, 5 mg/kg | 0.43 | 0.80 | 3.1 ± 1.2 | 22 | 111 |
| **2**, 8 mg/kg | 0.38 | 0.71 | 4.4 ± 1.5 | 20 | 100 |
| **3**, 6 mg/kg | 0.36 | 0.67 | 4.3 ± 2.3 | 22 | 111 |
| **4**, 5 mg/kg | 0.02 | 0.87 | 4.2 ± 1.9 | 22 | 111 |
| **5**, 6 mg/kg | 1.18 | 0.43 | 3.1 ± 0.8 | 22 | 111 |
| cisplatin, 5.5 mg/kg | 0.18 | 0.73 | 5.9 ± 1.5 | | |
| cisplatin, 9 mg/kg | 0.66 | 0.39 | 5.3 ± 1.3 | | |
| carboplatin, 90 mg/kg | 1.00 | 0.52 | 5.8 ± 1.6 | | |

GDF: Growth Delay Factor, indicates the tumour doubling time gained by treatment.

$GDF = (TDT_{treated} - TDT_{control}) TDT_{control}$

T/C: relative tumour volume treated mice/relative tumour volume control mice

MLS = MedianLife Span

ILS : Increase inLife Span = (MLS treated mice)/(MLS untreated mice) x 100%.

### Conclusion

Many di- and triorganotin compounds have been found much more active than cis-platin *in vitro* against two human tumour cell lines, MCF-7, a mammary tumour, and WiDr, a colon carcinoma. *In vivo* test results show that di-n-butyltin bis(2,5-dihydroxybenzoate), compound **5**, is comparably active to cisplatin at comparable doses. More work has however to be performed in order to find organotin molecules that might become useful antitumour drugs in the future.

### Acknowledgements

I would like to thank all my coworkers who prepared, purified and characterized the compounds reported in this paper, and also especially Prof. Dr. R. Willem, Dr. M. Biesemans and Dr. A. Delmotte who supervised this work. We also indebted to Dr. D. de Vos, Mr. H. J. Kolker, Dr. J. Verweij, Prof. Dr. G. Stoter, Dr. J. H. M. Schellens, Dr. C.M. Kuiper, Dr. G. Veerman and Prof. Dr. G. J. Peters for the *in vitro* and *in vivo* tests. This research was supported by the Belgian "Nationaal Fonds voor Wetenschappelijk Onderzoek" N.F.W.O. (grant number S2/5 CD F198, M.G.), and by the Human Capital and Mobility Programme of the European Community (Contract Nr ERBCHRXCT920016).

### References

(1) M. Bouâlam, M. Gielen, A. Meriem, D. de Vos and R. Willem (Pharmachemie B.V.): Anti-tumour compositions and compounds, *Eur. Pat.* 90202316.7-, 21/09/90; M. Bouâlam, M. Gielen, A. El Khloufi, D. de Vos and R. Willem, Pharmachemie B.V., Novel organo-tin compounds having anti-tumour activity and anti-tumour compositions, *Eur. Pat.* 91202746.3-, 22/10/91

(2) M. Gielen, P. Lelieveld, D. de Vos and R. Willem, *In vitro* antitumour activity of organotin compounds, in "Metal-Based Antitumour Drugs", vol. 2, Ed.: M. Gielen, Freund Publ. House, Tel Aviv, 1992, pp. 29 - 54 ; M. Gielen. P. Lelieveld, D. de Vos and R. Willem, "*In vitro* Antitumour Activity of Organotin(IV) Derivatives of Salicylic Acid and Related Compounds", in "Metal Complexes in Cancer Chemotherapy", Ed.: B. Keppler, VCH (Weinheim, Germany), 1993, pp. 383 - 390

(3) M. Gielen, M. Acheddad and E.R.T. Tiekink, *Main Group Met. Chem.*, **16** (1993), 367

(4) M. Gielen, M. Acheddad, B. Mahieu and R. Willem, *Main Group Met. Chem.*, **14** (1991), 73

(5) M. Gielen, A. El Khloufi, M. Biesemans, R. Willem and J. Meunier-Piret, *Polyhedron*, **11** (1992), 1861

(6) J. Meunier-Piret, M. Bouâlam, R. Willem and M. Gielen, *Main Group Met. Chem.*, **16** (1993), 329

(7) M. Gielen, R. Willem, M. Biesemans, M. Bouâlam, A. El Khloufi and D. de Vos, *Appl. Organomet. Chem.*, **6** (1992), 287

(8) M. Gielen, R. Willem, A. Bouhdid, D. de Vos, C.M. Kuiper and G. Veerman, *In vivo*, in press

**Received: December 2, 1994 - Accepted: January 9, 1995 - Received in revised camera-ready format: January 10, 1995**

